# Supplementary material for: Resolving the full spectrum of human genome variation using Linked-Reads
Source: Genome Res. 2019 Apr;29(4):635–45. doi: 10.1101/gr.234443.118 (PMC6442396; doi:10.1101/gr.234443.118)
Supplement: Supplemental Material [file supp_gr.234443.118_Supplemental_longranger-2.2.zip › longranger-2.2.tar.bz2!/lr2.2-public/lib/stan/cmdstan/stan/doc/api/html/agrad_2fwd_2matrix_2typedefs_8hpp_source.html]

Stan: src/stan/agrad/fwd/matrix/typedefs.hpp Source File


|  |  |
| --- | --- |
|  | Stan  2.5.0  probability, sampling & optimization |


- Main Page
- Related Pages
- Namespaces
- Classes
- Files

- File List
- File Members

All Classes Namespaces Files Functions Variables Typedefs Enumerations Enumerator Friends Macros Pages

- src
- stan
- agrad
- fwd
- matrix

typedefs.hpp

Go to the documentation of this file.

1 #ifndef STAN\_\_AGRAD\_\_FWD\_\_MATRIX\_\_TYPEDEFS\_HPP

2 #define STAN\_\_AGRAD\_\_FWD\_\_MATRIX\_\_TYPEDEFS\_HPP

3

4 #include <stan/math/matrix/Eigen.hpp>

5 #include <stan/agrad/fwd/fvar.hpp>

6 #include <stan/agrad/rev/var.hpp>

7 #include <stan/agrad/fwd/numeric\_limits.hpp>

8

9 namespace stan {

10  namespace agrad {

11

12  typedef

13  Eigen::Matrix<double,Eigen::Dynamic,Eigen::Dynamic>::Index

14  size\_type;

15

16  typedef

17  Eigen::Matrix<fvar<double>,Eigen::Dynamic,Eigen::Dynamic>

18  matrix\_fd;

19

20  typedef

21  Eigen::Matrix<fvar<var>,Eigen::Dynamic,Eigen::Dynamic>

22  matrix\_fv;

23

24  typedef

25  Eigen::Matrix<fvar<fvar<double> >,Eigen::Dynamic,Eigen::Dynamic>

26  matrix\_ffd;

27

28  typedef

29  Eigen::Matrix<fvar<fvar<var> >,Eigen::Dynamic,Eigen::Dynamic>

30  matrix\_ffv;

31

32  typedef

33  Eigen::Matrix<fvar<double>,Eigen::Dynamic,1>

34  vector\_fd;

35

36  typedef

37  Eigen::Matrix<fvar<var>,Eigen::Dynamic,1>

38  vector\_fv;

39

40  typedef

41  Eigen::Matrix<fvar<fvar<double> >,Eigen::Dynamic,1>

42  vector\_ffd;

43

44  typedef

45  Eigen::Matrix<fvar<fvar<var> >,Eigen::Dynamic,1>

46  vector\_ffv;

47

48  typedef

49  Eigen::Matrix<fvar<double>,1,Eigen::Dynamic>

50  row\_vector\_fd;

51

52  typedef

53  Eigen::Matrix<fvar<var>,1,Eigen::Dynamic>

54  row\_vector\_fv;

55

56  typedef

57  Eigen::Matrix<fvar<fvar<double> >,1,Eigen::Dynamic>

58  row\_vector\_ffd;

59

60  typedef

61  Eigen::Matrix<fvar<fvar<var> >,1,Eigen::Dynamic>

62  row\_vector\_ffv;

63

64  }

65 }

66 #endif

stan::agrad::row\_vector\_fd

Eigen::Matrix< fvar< double >, 1, Eigen::Dynamic > row\_vector\_fd

**Definition:** typedefs.hpp:50

stan::agrad::row\_vector\_ffv

Eigen::Matrix< fvar< fvar< var > >, 1, Eigen::Dynamic > row\_vector\_ffv

**Definition:** typedefs.hpp:62

stan::agrad::row\_vector\_fv

Eigen::Matrix< fvar< var >, 1, Eigen::Dynamic > row\_vector\_fv

**Definition:** typedefs.hpp:54

numeric\_limits.hpp

stan::agrad::size\_type

Eigen::Matrix< double, Eigen::Dynamic, Eigen::Dynamic >::Index size\_type

**Definition:** typedefs.hpp:14

stan::agrad::vector\_fd

Eigen::Matrix< fvar< double >, Eigen::Dynamic, 1 > vector\_fd

**Definition:** typedefs.hpp:34

stan::agrad::row\_vector\_ffd

Eigen::Matrix< fvar< fvar< double > >, 1, Eigen::Dynamic > row\_vector\_ffd

**Definition:** typedefs.hpp:58

fvar.hpp

var.hpp

Eigen.hpp

stan::agrad::matrix\_fd

Eigen::Matrix< fvar< double >, Eigen::Dynamic, Eigen::Dynamic > matrix\_fd

**Definition:** typedefs.hpp:18

stan::agrad::matrix\_ffv

Eigen::Matrix< fvar< fvar< var > >, Eigen::Dynamic, Eigen::Dynamic > matrix\_ffv

**Definition:** typedefs.hpp:30

stan::agrad::vector\_fv

Eigen::Matrix< fvar< var >, Eigen::Dynamic, 1 > vector\_fv

**Definition:** typedefs.hpp:38

stan::agrad::matrix\_fv

Eigen::Matrix< fvar< var >, Eigen::Dynamic, Eigen::Dynamic > matrix\_fv

**Definition:** typedefs.hpp:22

stan::agrad::vector\_ffd

Eigen::Matrix< fvar< fvar< double > >, Eigen::Dynamic, 1 > vector\_ffd

**Definition:** typedefs.hpp:42

stan::agrad::vector\_ffv

Eigen::Matrix< fvar< fvar< var > >, Eigen::Dynamic, 1 > vector\_ffv

**Definition:** typedefs.hpp:46

stan::agrad::matrix\_ffd

Eigen::Matrix< fvar< fvar< double > >, Eigen::Dynamic, Eigen::Dynamic > matrix\_ffd

**Definition:** typedefs.hpp:26


---

[ Stan Home Page ]

*© 2011–2014,
Stan Development Team.*
